# Supplementary material for: Decoration of Polyfluorene-Wrapped Carbon Nanotubes with Photocleavable Side-Chains
Source: Molecules. 2023 Feb 3;28(3):1471. doi: 10.3390/molecules28031471 (PMC9920975; doi:10.3390/molecules28031471)
Supplement: Supplementary file 1 [file molecules-28-01471-s001.zip › molecules-2197898-supplementary.pdf]

SUPPORTING INFORMATION

Decoration of polyfluorene-wrapped carbon nanotubes using photocleavable cleavable side chains

Dialia Ritaine, Alex Adronov*

Department of Chemistry and Chemical Biology and the Brockhouse Institute for Materials Research, McMaster University, 1280 Main St. W., Hamilton, Ontario, Canada L8S 4M1

Experimental

Synthetic procedures

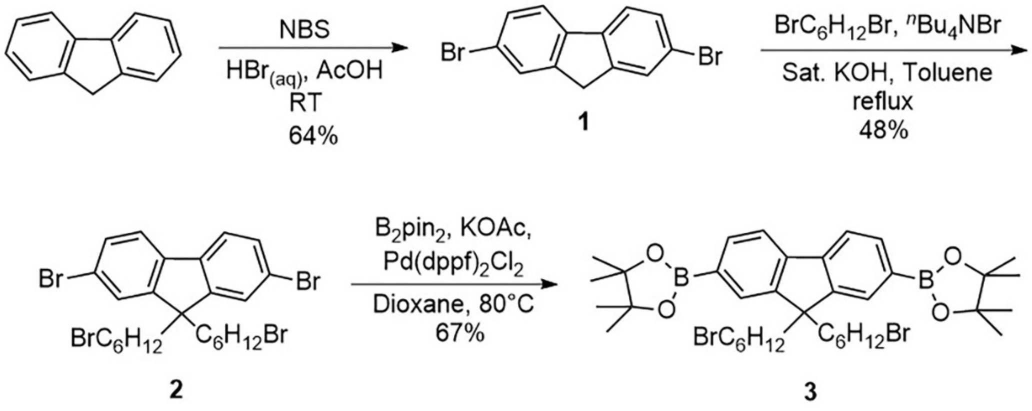

Scheme S1: Synthesis of monomers **2** and **3** [1].

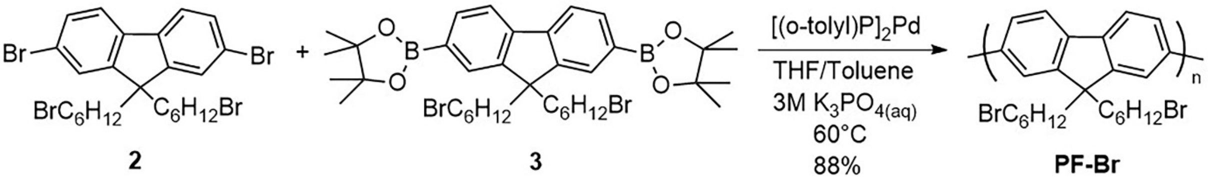

Scheme S2: Synthesis of **PF-Br** [1].

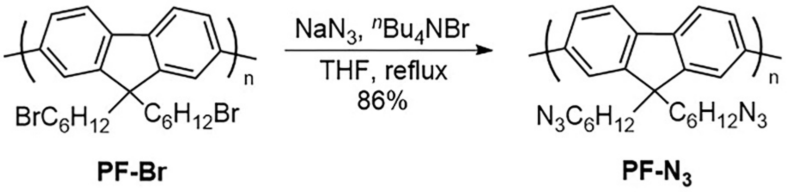

Scheme S3: Synthesis of **PF-N$_3$** [1].

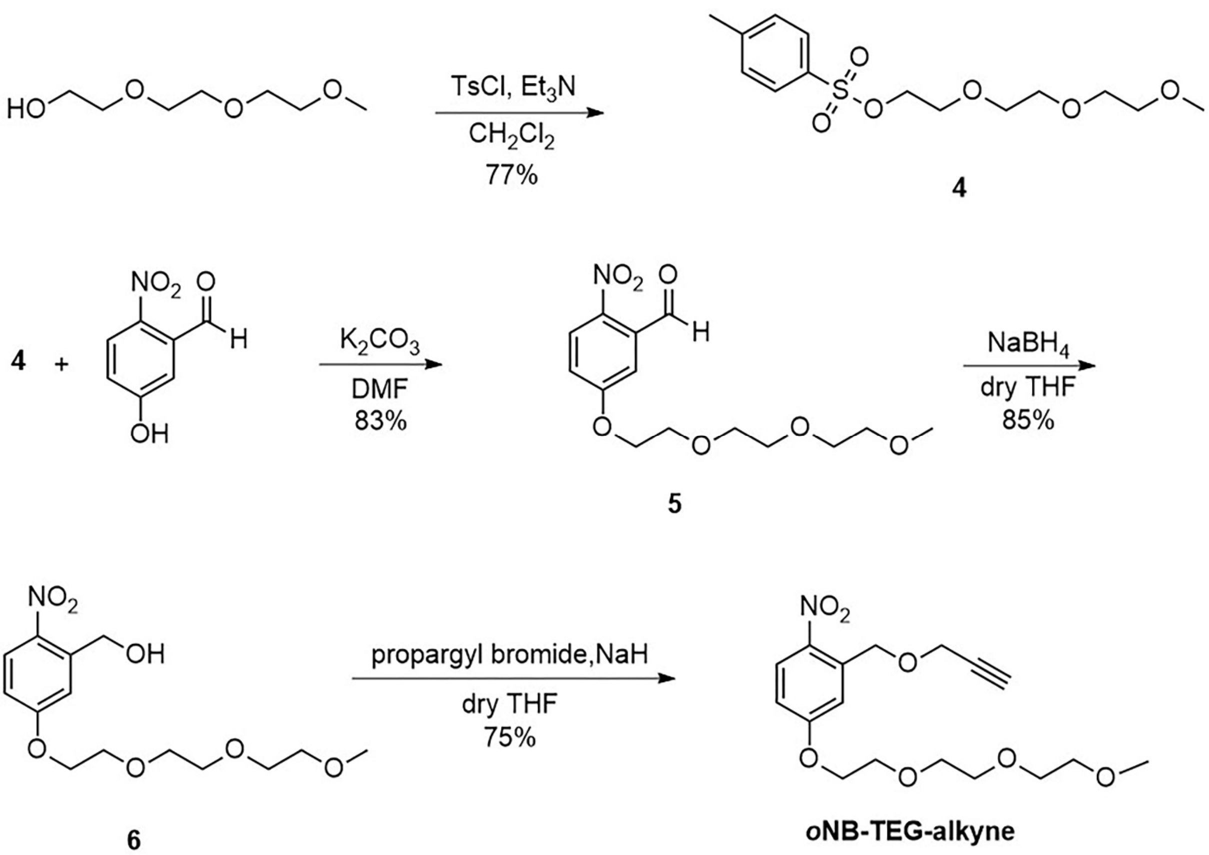

Scheme S4: Synthesis of ***o*NB-TEG-alkyne** [2,3]**.**

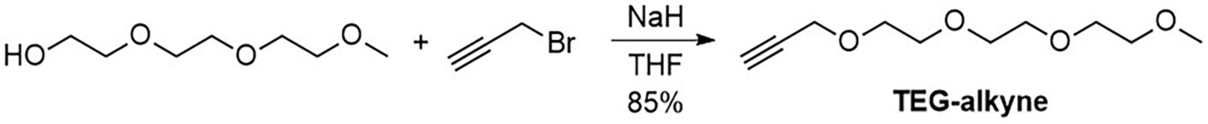

Scheme S5: Synthesis of **TEG-alkyne** [4].

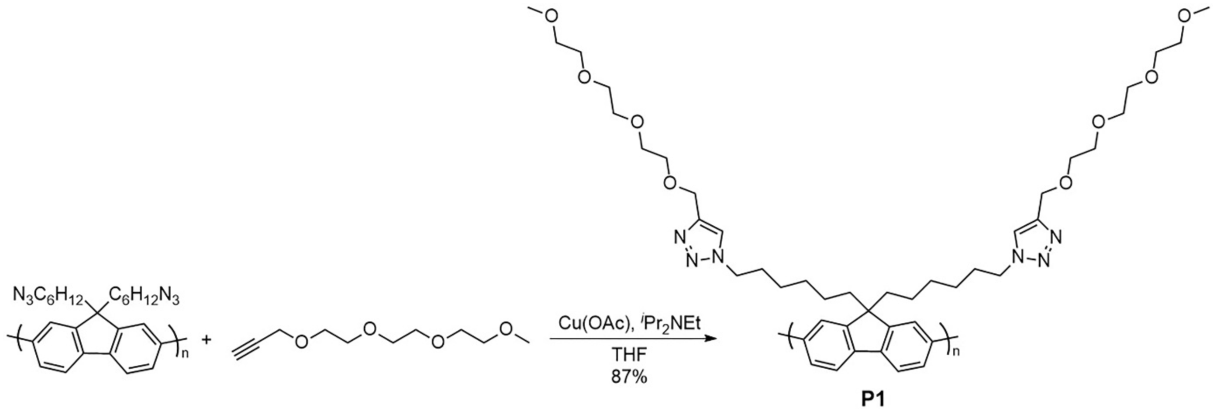

Scheme S6: Synthesis of **PF-TEG (P1)**.

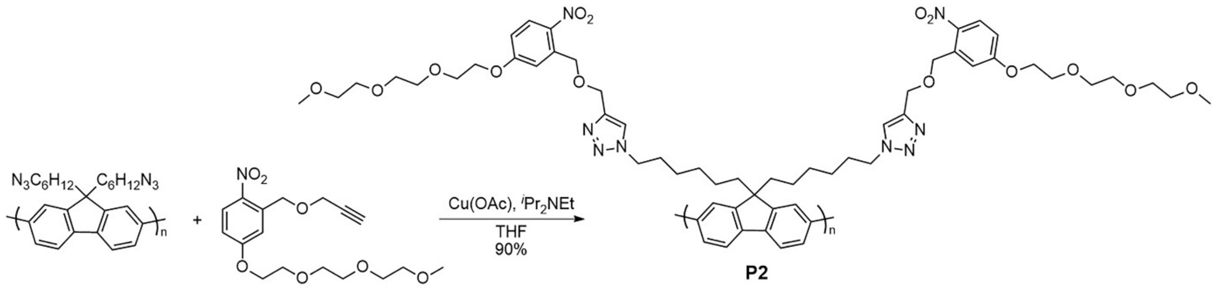

Scheme S7: Synthesis of **PF-*o*NB-TEG (P2)**.

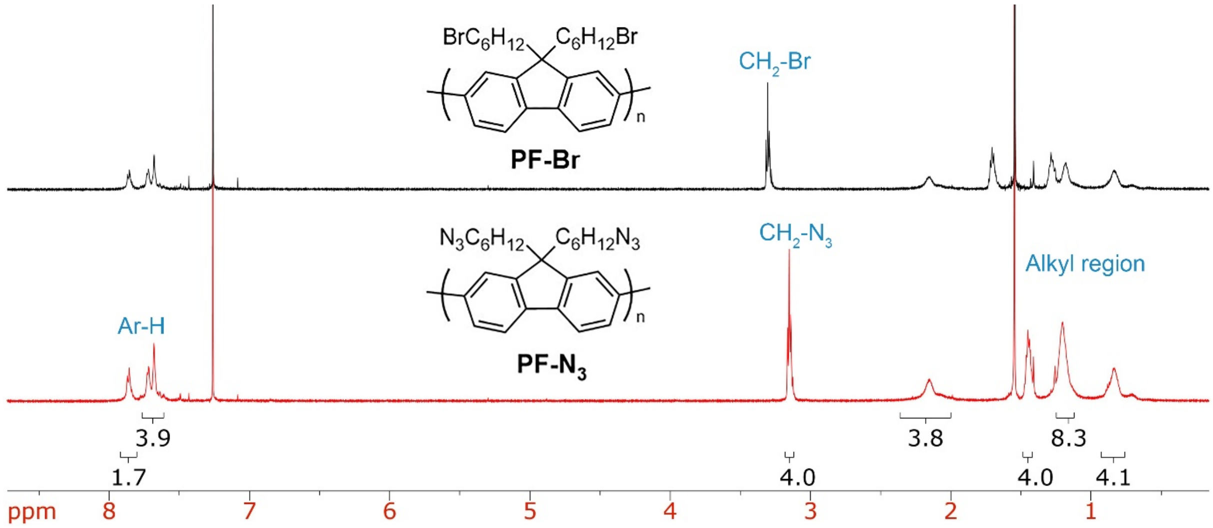

Figure S1. ^{1}H NMR overlay of **PF-Br** (top) and **PF-N$_3$** (bottom).

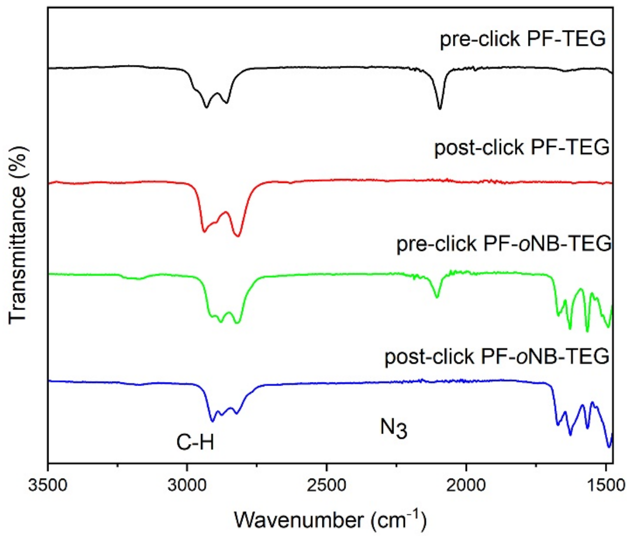

Figure S2. FT-IR overlay of the click reaction between **PF-N₃**, TEG-alkyne and *o*NB-TEG-alkyne.

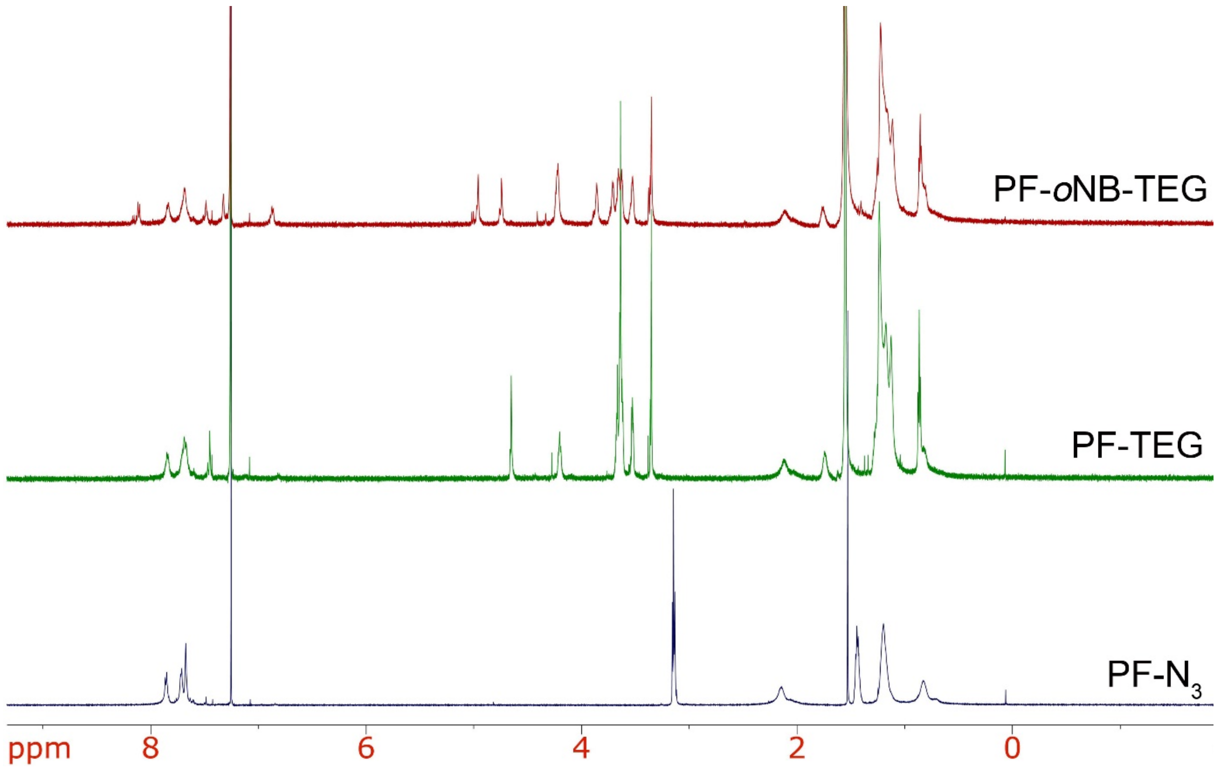

Figure S3: ^{1}H NMR overlay of **PF-N$_3$** (bottom), **PF-TEG** (middle) and **PF-oNB-TEG** (top).

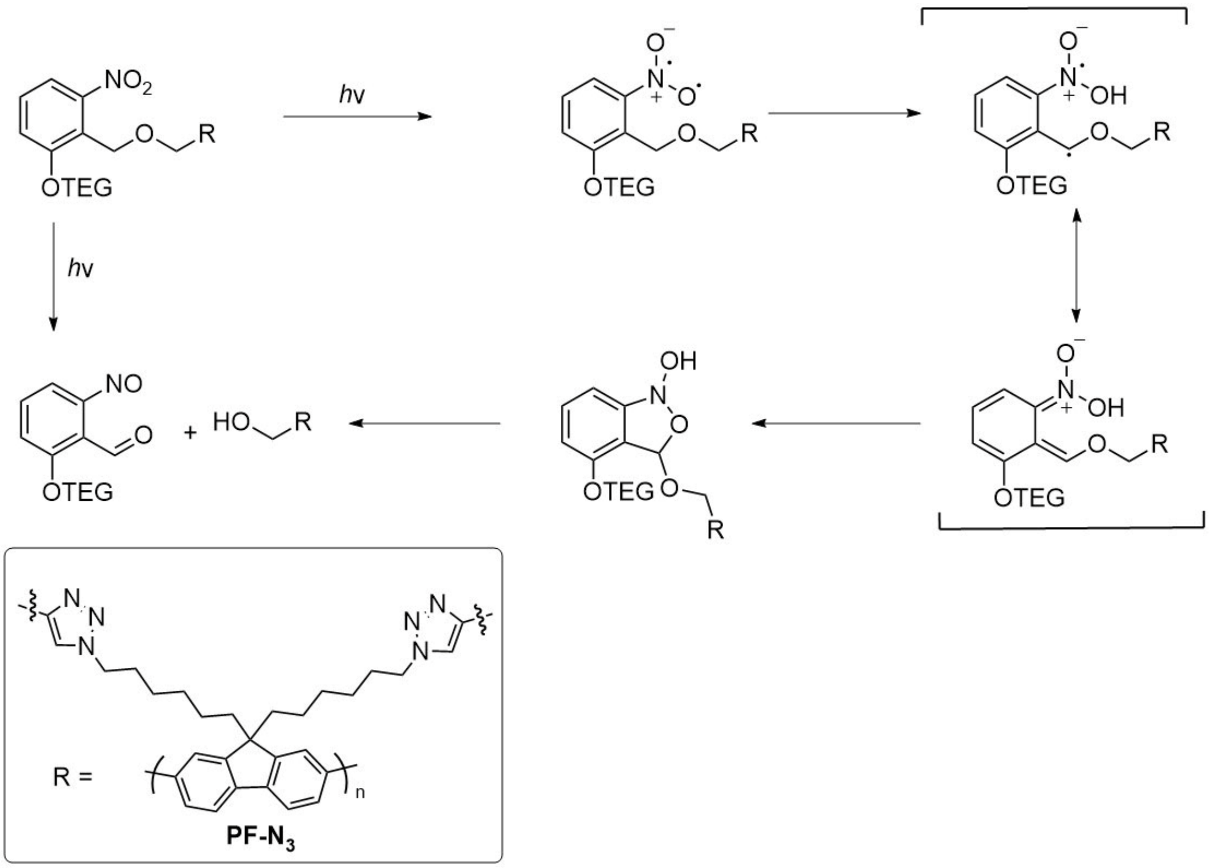

Figure S4: Photoisomerization mechanism of **PF**-*o*NB-TEG. Adapted from reference [2]

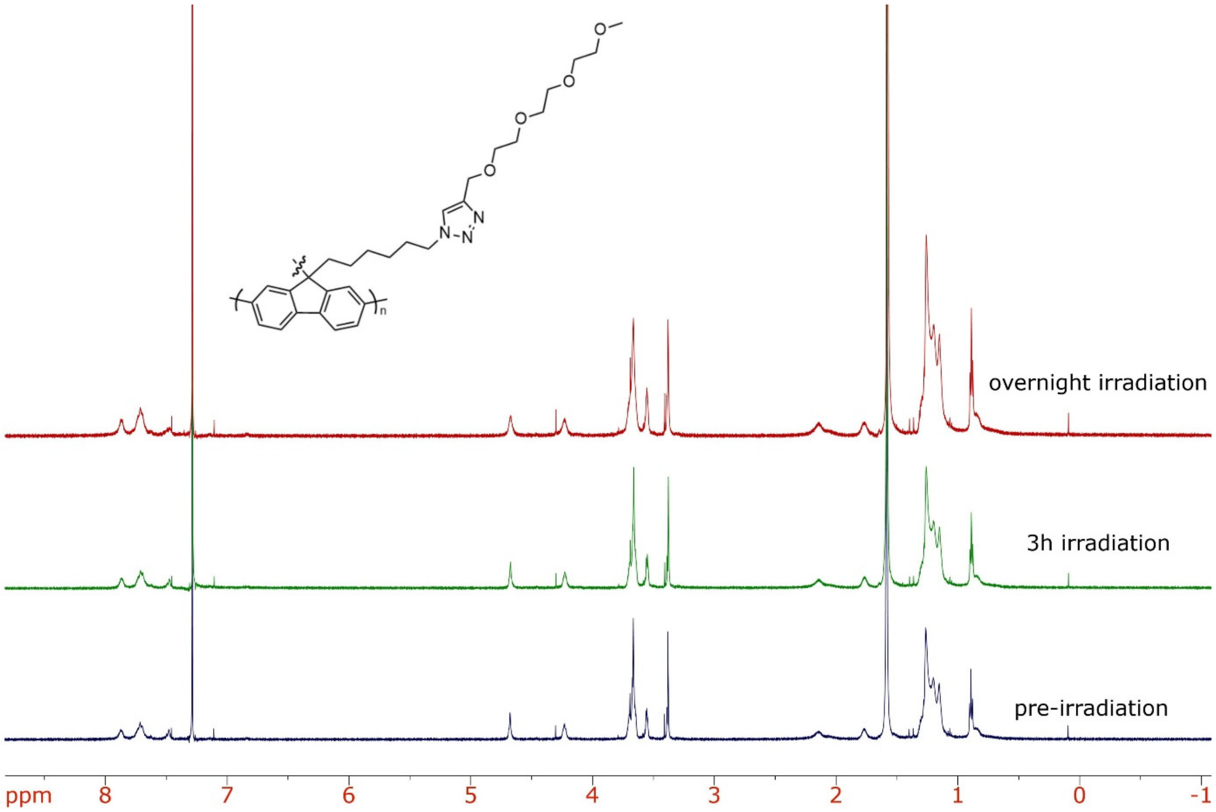

Figure S5. ^{1}H NMR overlay of **PF-TEG** before irradiation (bottom), after 3 hours of irradiation (middle) and after overnight irradiation (top).

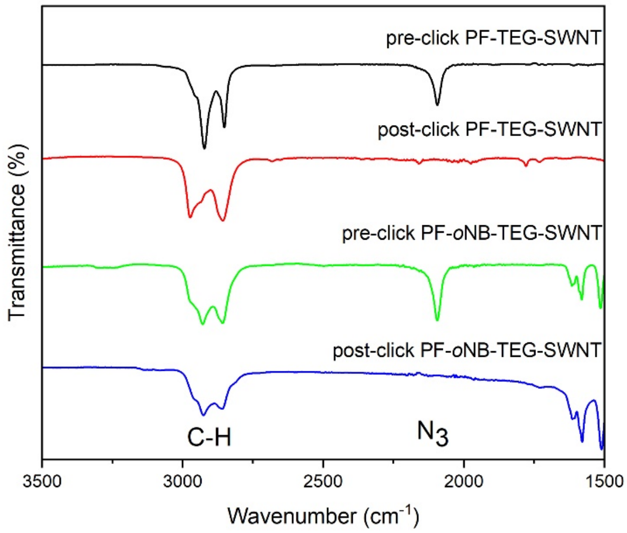

Figure S6. FT-IR overlay of the click reaction between the **PF-N$_3$**-SWNT complex, TEG-alkyne and *o*NB-TEG-alkyne.

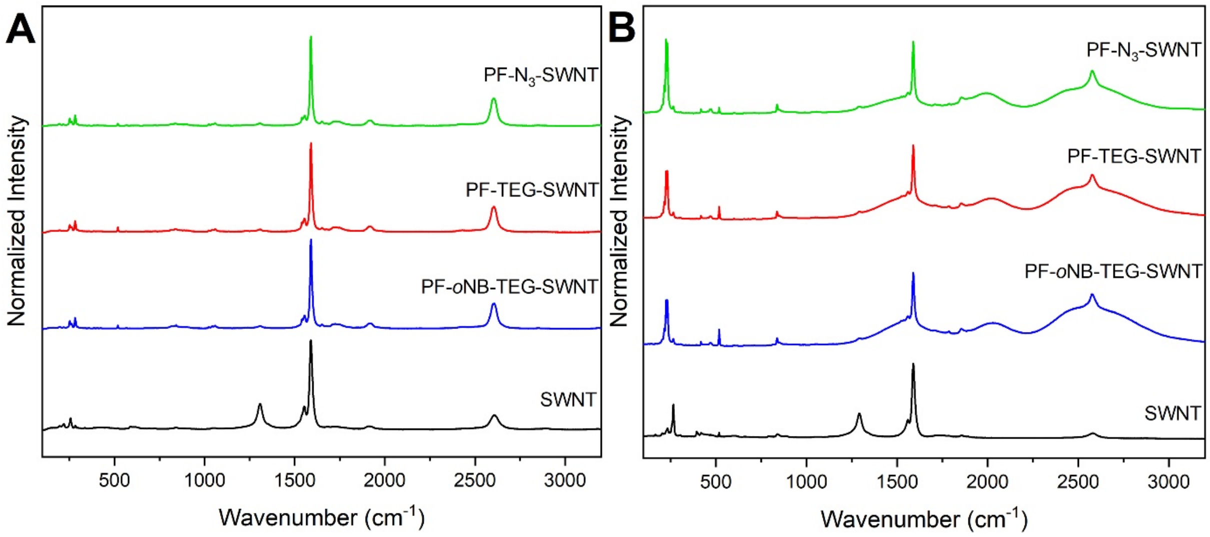

Figure S7: Full Raman spectra for HipCo polymer-SWNT samples at A) λ_{ex} = 633 nm, B) λ_{ex} = 785 nm. All the spectra were normalized at ~ 1590 cm^{-1}.

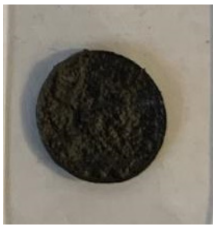

Figure S8. Photograph of a **PF**-SWNT thin film.

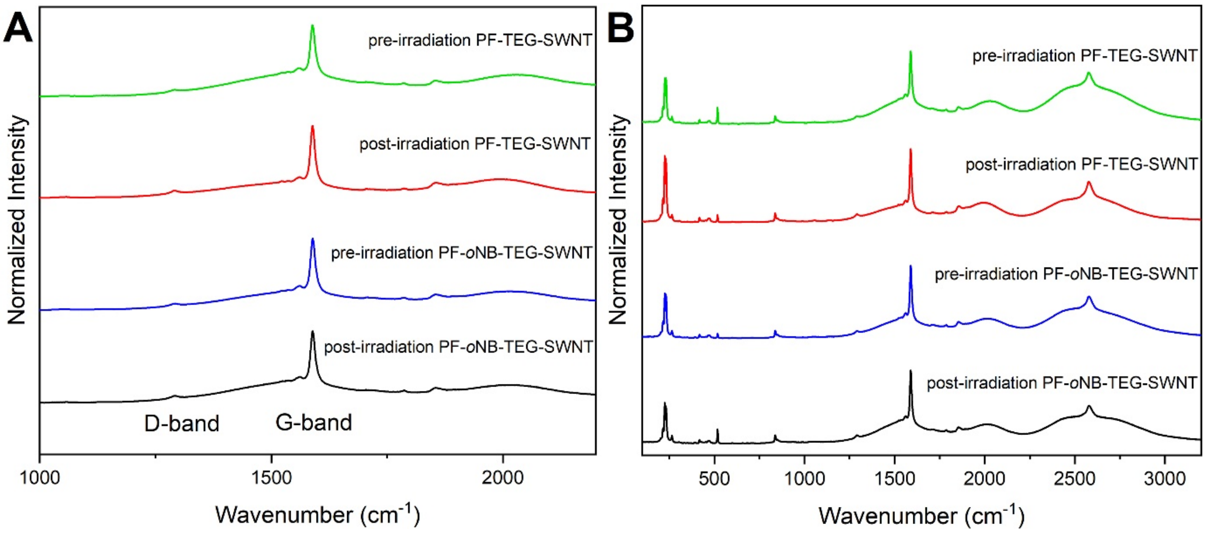

Figure S9. Full Raman spectra for **PF-TEG-SWNT** and **PF-*o*NB-TEG-SWNT** pre- and post-irradiation at A) λ_{ex} = 785 nm. All the spectra were normalized at ~ 1590 cm^{-1}.

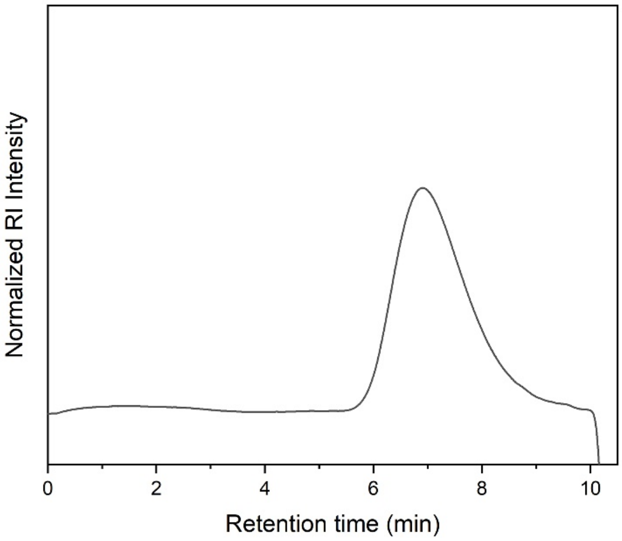

Figure S10. GPC trace of **PF-Br**.

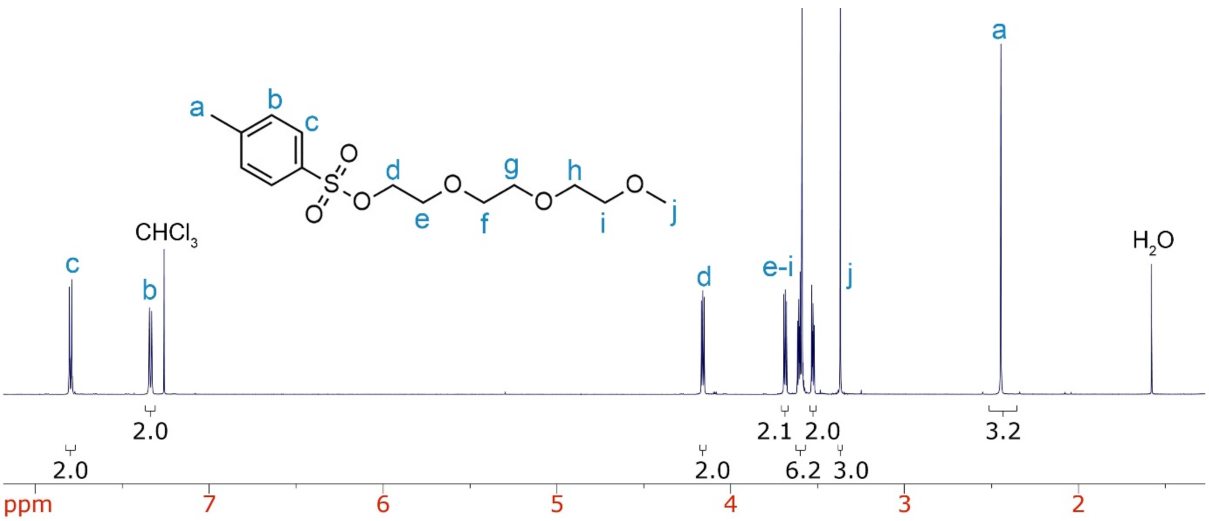

Figure S11. ^{1}H NMR spectrum of compound **4**.

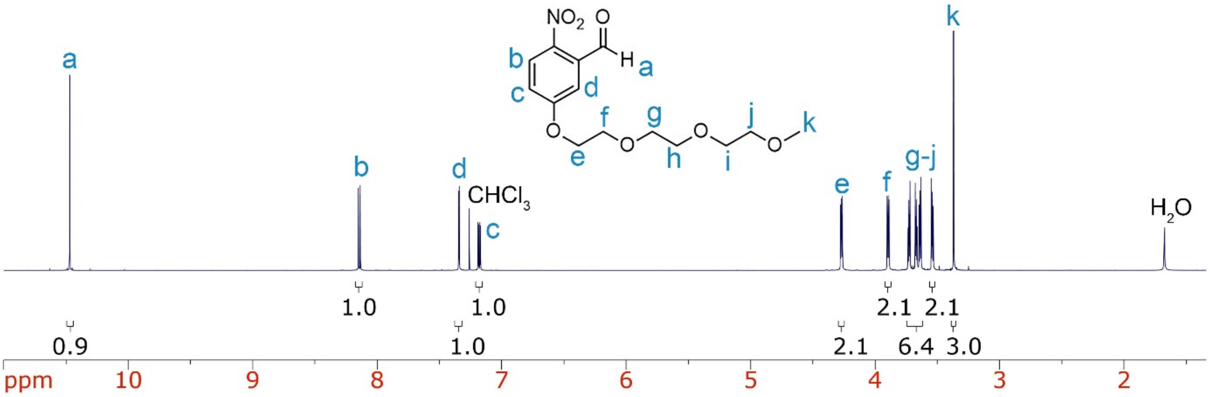

Figure S12. ^{1}H NMR spectrum of compound **5**.

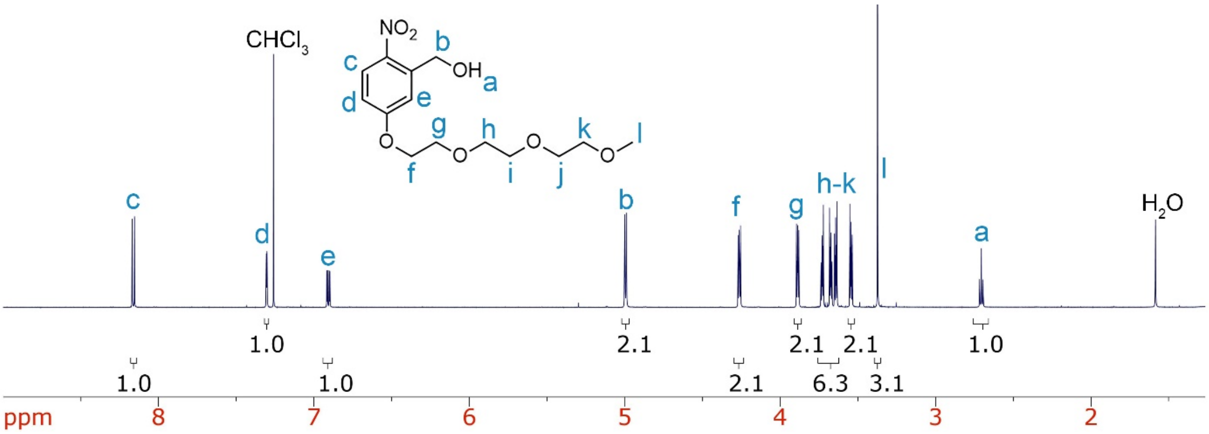

Figure S13. ^{1}H NMR of compound **6**.

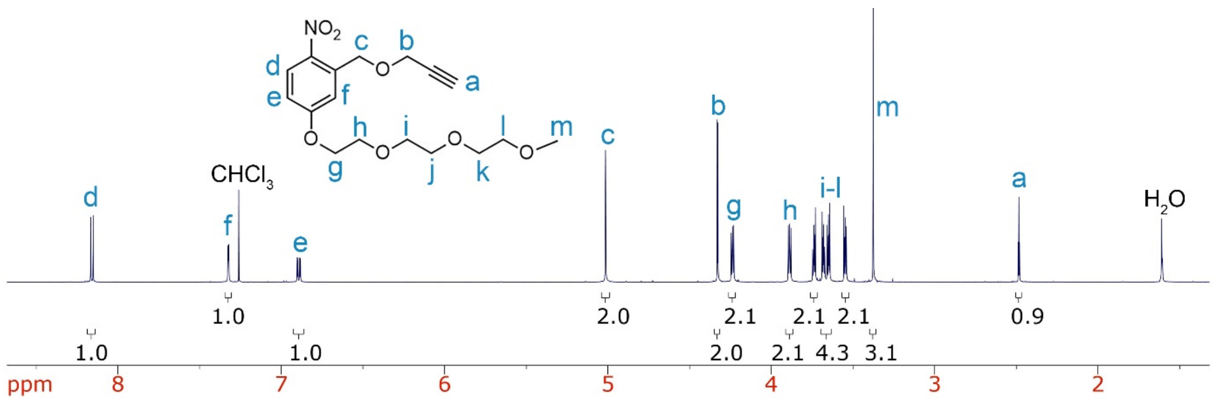

Figure S14a. ^{1}H NMR spectrum of ***o*NB-TEG-alkyne**.

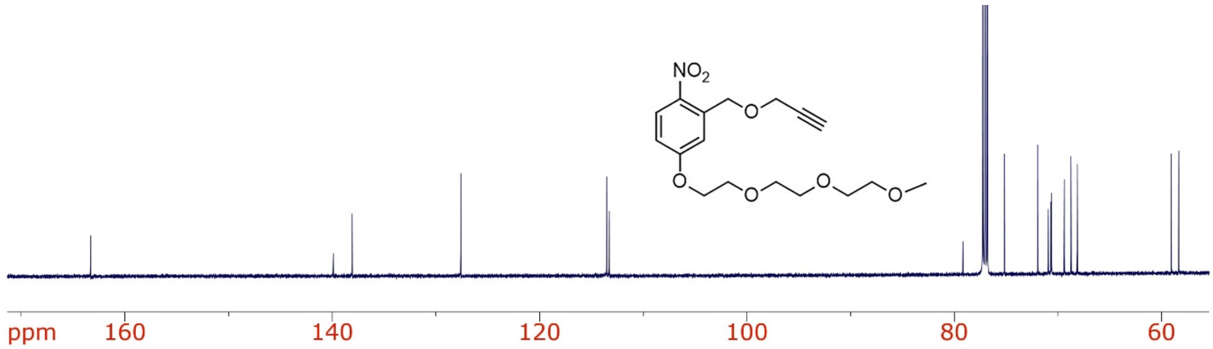

Figure S14b. ^{13}C NMR spectrum of compound ***o*NB-TEG-alkyne** in $CDCl_3$.

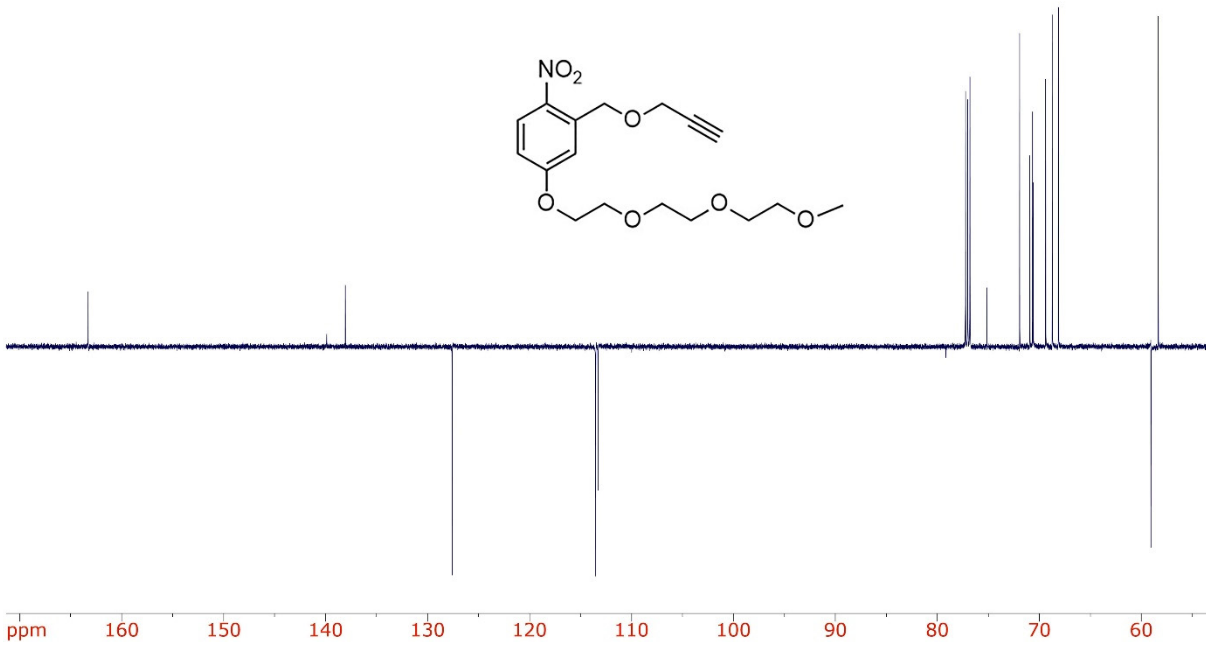

Figure S14c. Deptq NMR of compound ***o*****NB-TEG-alkyne** $CDCl_3$.

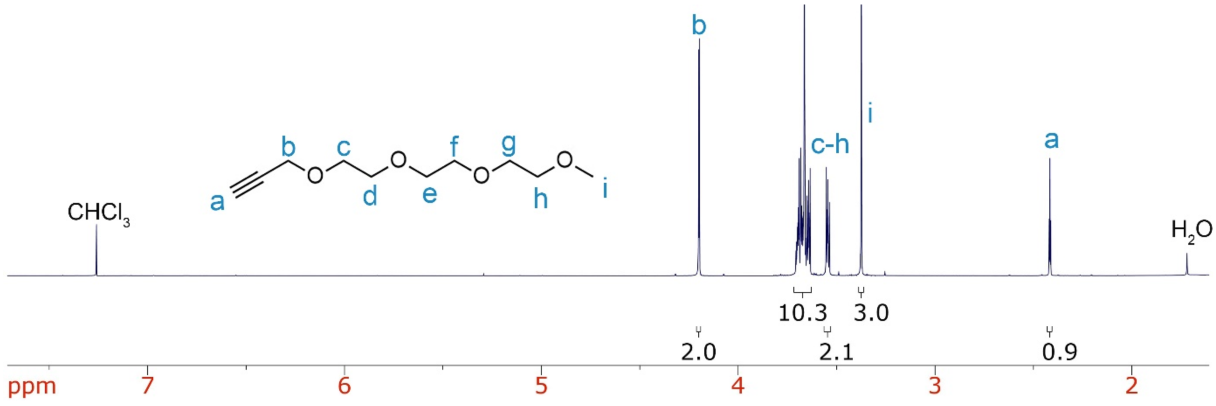

Figure S15. ^{1}H NMR spectrum of **TEG-alkyne**.

1. Fong, D.; Yeung, J.; Meichsner, E.; Adronov, A. Reactive, Aqueous-Dispersible Polyfluorene-Wrapped Carbon Nanotubes Modulated with an Acidochromic Switch via Azide–Alkyne Cycloaddition. *ACS Appl. Polym. Mater.* **2019**, *1*, 797–803, doi:10.1021/acsapm.9b00040.

2. Chan, E.W.C.; Baek, P.; Tan, S.M.; Davidson, S.J.; Barker, D.; Travas-Sejdic, J. Molecular "Building Block" and "Side Chain Engineering": Approach to Synthesis of Multifunctional and Soluble Poly(Pyrrole Phenylene)S. *Macromol. Rapid Commun.* **2019**, *40*, 1–6, doi:10.1002/marc.201800749.

3. Suzuki, Y.; Sakamoto, T.; Yoshio, M.; Kato, T. Development of Functional Nanoporous

Membranes Based on Photocleavable Columnar Liquid Crystals – Selective Adsorption of Ionic Dyes. *Eur. Polym. J.* **2020**, *134*, 109859, doi:10.1016/j.eurpolymj.2020.109859.

4. Chandra, P.; Jonas, A.M.; Fernandes, A.E. Sequence and Surface Confinement Direct Cooperativity in Catalytic Precision Oligomers. *J. Am. Chem. Soc.* **2018**, *140*, 5179–5184, doi:10.1021/jacs.8b00872.
